# Differences in essential newborn care at birth between private and public health facilities in eastern Uganda

**DOI:** 10.3402/gha.v8.24251

**Published:** 2015-03-31

**Authors:** Peter Waiswa, Joseph Akuze, Stefan Peterson, Kate Kerber, Moses Tetui, Birger C. Forsberg, Claudia Hanson

**Affiliations:** 1 Global Health, Department of Public Health Sciences, Karolinska Institutet, Stockholm, Sweden; 2 Department of Health Policy, Planning and Management, Makerere University School of Public Health, Kampala, Uganda; 3 Iganga-Mayuge Health Demographic Surveillance Site, Iganga-Mayuge, Uganda; 4 International Maternal and Child Health Unit, Department of Women's and Children's Health, Uppsala University, Uppsala, Sweden; 5 Saving Newborn Lives, Save the Children, Cape Town, South Africa

**Keywords:** newborn health, private health care, public health care, essential newborn care, Uganda

## Abstract

**Background:**

In Uganda and elsewhere, the private sector provides an increasing and significant proportion of maternal and child health services. However, little is known whether private care results in better quality services and improved outcomes compared to the public sector, especially regarding care at the time of birth.

**Objective:**

To describe the characteristics of care-seekers and assess newborn care practices and services received at public and private facilities in rural eastern Uganda.

**Design:**

Within a community-based maternal and newborn care intervention with health systems strengthening, we collected data from mothers with infants at baseline and endline using a structured questionnaire. Descriptive, bivariate, and multivariate data analysis comparing nine newborn care practices and three composite newborn care indicators among private and public health facilities was conducted.

**Results:**

The proportion of women giving birth at private facilities decreased from 25% at baseline to 17% at endline, whereas overall facility births increased. Private health facilities did not perform significantly better than public health facilities in terms of coverage of any essential newborn care interventions, and babies were more likely to receive thermal care practices in public facilities compared to private (68% compared to 60%, *p*=0.007). Babies born at public health facilities received an average of 7.0 essential newborn care interventions compared to 6.2 at private facilities (*p*<0.001). Women delivering in private facilities were more likely to have higher parity, lower socio-economic status, less education, to seek antenatal care later in pregnancy, and to have a normal delivery compared to women delivering in public facilities.

**Conclusions:**

In this setting, private health facilities serve a vulnerable population and provide access to service for those who might not otherwise have it. However, provision of essential newborn care practices was slightly lower in private compared to public facilities, calling for quality improvement in both private and public sector facilities, and a greater emphasis on tracking access to and quality of care in private sector facilities.

Newborn mortality (deaths within the first 28 days of life) remains unacceptably high in sub-Saharan Africa and in Uganda. Every year 2.9 million babies die during the neonatal period (1), with the majority of these deaths occurring in the first week of life. This is also the time of greatest risk for stillbirths and maternal deaths (2). In Uganda, out of 1.5 million births in 2012, 82,000 resulted in a mother or baby dying (3). There are many missed opportunities to improve care and increase the potential to save lives at and immediately after birth. Saving mothers and babies is rarely the result of a single, simple intervention, but a complex and comprehensive set of interlinked services and practices supported by health workers.

Skilled attendance at birth is considered a critically important platform to reduce the burden of maternal and newborn mortality worldwide (4, 5). If backed by a referral level providing comprehensive emergency obstetric care, uptake of skilled attendance will prevent by far the largest part of maternal and newborn mortality as well as many stillbirths (2). However, skilled attendance will only have the promised effect if the different components of it are implemented, and thus quality of care is assured (6). Although quality of care is a complex and multidimensional concept including safety, effectiveness, timeliness, efficiency, equity, and patient-centredness (7), measuring signal indicators may provide some insight into the coverage and quality of care overall (8).

Private for-profit providers, typically small privately owned clinics with a single proprietor, play a significant role in provision of outpatient health care and reproductive health, but involvement in maternity care is a relatively recent phenomenon in sub-Saharan Africa, where most women still deliver at home or choose the public health system (9). Still, in some countries such as Nigeria or Kenya, a larger proportion of deliveries are now taking place in private clinics and hospitals (10–13). One of the milestones of the newly launched Every Newborn Action Plan is coordinated support and effort amongst private sector providers of delivery services and newborn care (14).

Engagement of the private sector to increase accessibility to reproductive and child health care is much debated (15). Some studies have reported that greater participation of the private sector improved access to and equity in care (16, 17), whereas others indicated the opposite (18). Criticisms in regard to private maternity care include late referral to public health facilities in the case of obstetric emergencies, as private maternity facilities are not always equipped to provide 24-h emergency obstetric care services (19, 20). Where operative services are available, the fees charged for a caesarean section delivery might increase caesarean section rates, particularly where third-party fee-for-service reimbursement gives health providers an income from their services.

Families often seek out private health facilities as they perceive the quality of care as better overall (21–28), although a recent systematic review suggests that quality in both public and private provider groups is poor, with the private sector being better in terms of drug availability and aspects of responsiveness to client expectations (29). Results from an investigation into the use of private maternity services from a Nairobi informal settlement indicated that private care was less costly, closer to the home, and providers were more empathic (30, 31). In Nigeria, private maternity care was the preferred place of delivery because of the low quality of government facilities, particularly with respect to absence of staff, poor perceived quality, waiting times, and high costs (11).

Despite increasing prominence of the private sector as a provider of delivery and newborn care, there is a dearth of data on newborn care practices in these facilities. To the best of our knowledge, no information on the quality of delivery care of private providers is available from Uganda. As part of the Uganda Newborn Study (UNEST) (32), we engaged public as well as private for-profit and not-for-profit providers through sensitisation, training, and supervision around childbirth and newborn care. UNEST aimed at improving newborn survival through a community-based intervention using home visits by volunteers linked to health facilities. The intervention included a health system strengthening component and improving linkages between the community-based intervention and the health facilities. Here we present the determinants of use and the quality of public and private maternity care. Furthermore, we assessed the impact of facility strengthening on implementation of essential newborn care interventions among births that occurred in private and public health facilities in rural eastern Uganda. This is the sixth paper in a series on the UNEST results.

## Methods

### Study design and setting

**Fig. 1:**
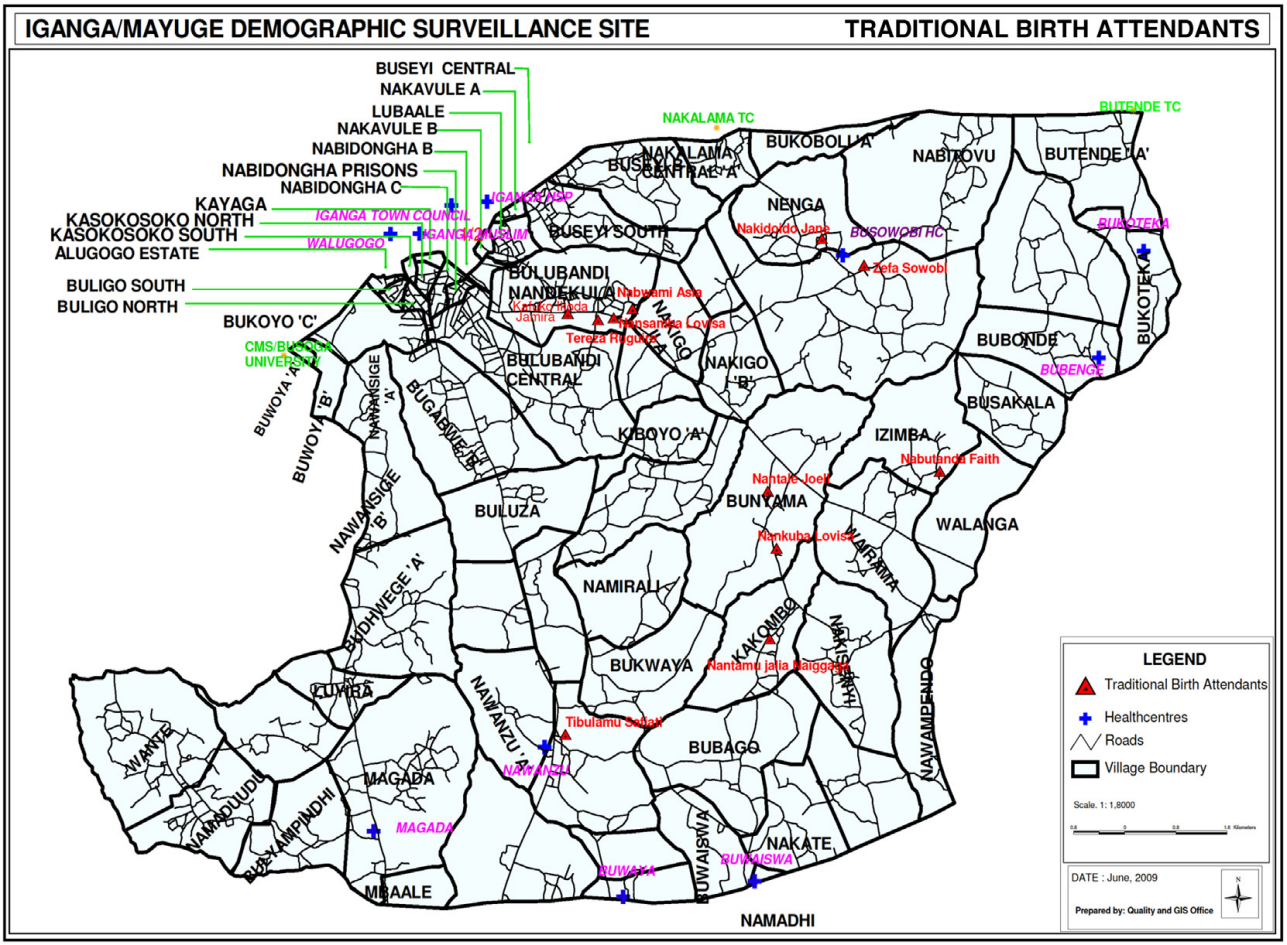
Map of the UNEST study area.

The UNEST design and package has been described elsewhere (32–34). In brief, the study took place in the Iganga-Mayuge Health and Demographic Surveillance Site (HDSS) located in Iganga and Mayuge districts in the eastern region of Uganda, about 120 km east of the capital city of Kampala. The HDSS serves a population size of 70,000 people, at the time of the study, living in 65 villages, with women of reproductive age comprising 23%. The total fertility rate of the HDSS is 4.3. The population is served by 20 facilities including six private facilities (Fig. 1). The public hospital in Iganga is the only comprehensive emergency obstetric care facility. The public facilities charge no fees for services, although there are often informal costs requested of families. Typically, private facilities consisted of a small clinic with less than five staff who could provide essential care for common conditions. Private facilities are more accessible to the population and sometimes to rural areas than public facilities.

Villages were randomised to intervention or control arms. Intervention villages had a community health worker who was trained to provide home visits during pregnancy and the first week after delivery, whereas comparison villages received the standard care as delivered by the facilities in the area. Health facility strengthening including training of health workers on essential maternal-newborn care skills and provision of medicine, basic equipment, and supplies was done in all health facilities with a reasonable client load (more than 15–20 per month) for delivery care, independent of ownership and management or whether the facility was located in the intervention or comparison area. Both public and private health facilities were supported by quarterly supervision as part of the health system strengthening. In addition, linkages between community and health facilities were strengthened.

### Data collection

A standardised tool was adapted and pretested for data collection. Data collectors were experienced HDSS field staff. The baseline census was done between March and August 2007. Women with infants aged 1–4 months (*n*=395) in the HDSS were interviewed through visits to all households (35). At endline census, done between August and November 2011, we interviewed all women of childbearing age who had had a live birth in the previous 12 months (*n*=1,761) (17, 36–38).

### Data analysis

All analyses used Stata software version 12.1. Univariate and bivariate analyses were used to describe background characteristics of women who delivered in a health facility. The chi-square test was used to compare the difference between the private and public facilities as place of delivery. A multiple logistical regression model was constructed to identify determinants of private facility births using all of the explanatory variables which were significant at bivariate analysis. We checked for multicollinearity between the independent variables, and only included non-collinear variables in the analysis. For this study the effect of treatment – overall and within subgroups – and covariates were reported using odds ratios (ORs).

Data on nine essential newborn care practices were collected. These interventions included wrapping the baby immediately after birth using a dry cloth, early skin-to-skin placement, delayed bath at least 6 h after delivery, clean instrument used to cut the umbilical cord, clean device used to tie or clamp the cord, placing nothing on the cord stump, breastfeeding within the first hour after birth; not giving the baby a bottle, and not giving any food or drink other than breast milk. Interventions were combined into composite indicators for thermal care, hygienic cord care, and optimal feeding practices. In addition we assessed how many women received more than one to all nine essential newborn care interventions.

**Fig. 2:**
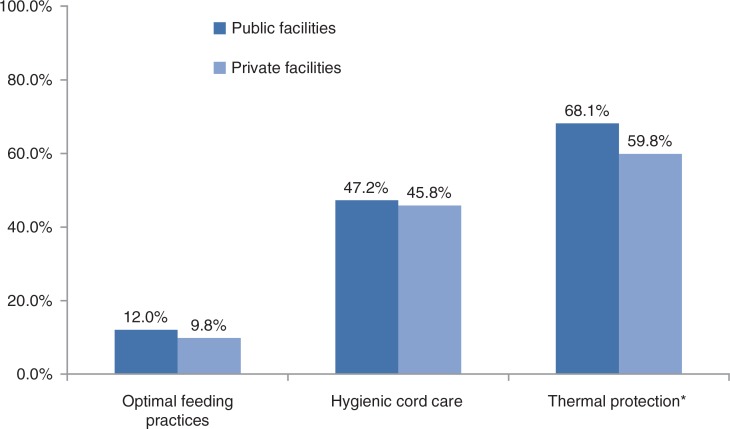
Coverage of babies receiving essential newborn care interventions. * χ^2^prob=0.007

Wealth quintiles were constructed using the Principal Component Analysis based on household assets as used by the Ugandan Bureau of Statistics, including number of sleeping rooms, type of floor material, type of roof material, wall material, type of bed, fuel used for cooking, source of light; and possession of a radio, a sewing machine, an electric flat iron, charcoal flat iron, a bed net, kerosene lamp, kerosene stove, car, tea table, refrigerator, television set, sound stereo, telephone, mattress, wheelbarrow, cell phone, and camera. These gave a Cronbach's alpha of 0.848. Principal component analysis was performed and the first principal component was scored to create an asset index that was used to group all households in the HDSS into wealth quintiles (35). Schooling was assessed using categories of completed education level.

## Results

### Background characteristics

The average age of the women who delivered in a health facility was 26 years, with no significant difference between private and public sector (Table 1). Nine of 10 mothers were married. Slightly more than half of all women had primary education as the highest level attained, and 9% had no education at all. More than one-third of women (38%) had given birth four or more times. Almost all women (99%) attended at least one antenatal care (ANC) visit and 49% attended four or more times. Less than one-fourth (23.6%) of women attended ANC in their first trimester. The rate of caesarean section was 4.4% overall.

**Table 1:** Background characteristics of respondents for endline census

	Total (all facility deliveries)	Private facilities	Public facilities	

Characteristics	N (%)	N (%)	N (%)	*p*
Maternal age (yrs)	*n*=1,358	*n*=299	*n*=1,059	
<19	96 (7.07)	16 (5.35)	80 (7.55)	0.1797
19–25	564 (41.53)	120 (40.13)	444 (41.93)	0.5770
26–30	357 (26.29)	79 (26.42)	278 (26.25)	0.9530
>30	341 (25.11)	84 (28.09)	257 (24.27)	0.1786
Marital status	*n*=1,369	*n*=306	*n*=1,063	
Not married	116 (8.47)	22 (7.19)	94 (8.84)	0.3610
Married	1,253 (91.53)	284 (92.81)	969 (91.16)	0.3610
Education	*n*=1,369	*n*=306	*n*=1,063	
No education	127 (9.28)	33 (10.78)	94 (8.84)	0.3026
Primary	783 (57.20)	198 (64.71)	585 (55.03)	0.0026*
Secondary	407 (29.73)	68 (22.22)	339 (31.89)	0.0011*
University	52 (3.80)	7 (2.29)	45 (4.23)	0.1176
Wealth quintile	*n*=1,036	*n*=240	*n*=796	
1 (Poorest)	154 (14.86)	46 (19.17)	108 (13.57)	0.0326*
2 (Poor)	219 (21.14)	52 (21.67)	167 (20.98)	0.8185
3 (Average)	258 (24.90)	63 (26.25)	195 (24.50)	0.5827
4 (Rich)	207 (19.98)	48 (20.00)	159 (19.97)	0.9919
5 (Richest)	198 (19.11)	31 (12.92)	167 (20.98)	0.0054*
Parity	*n*=1,369	*n*=306	*n*=1,063	
1	262 (19.14)	37 (12.09)	225 (21.17)	<0.001
2–4	585 (42.73)	129 (42.16)	456 (42.90)	0.8176
>4	522 (38.13)	140 (45.75)	382 (35.94)	0.0019*
Number of ANC visits	*n*=1,351	*n*=300	*n*=1,051	
1	46 (3.40)	15 (5.00)	31 (2.95)	0.0842
2–3	646 (47.82)	142 (47.33)	504 (47.95)	0.8496
>3	659 (48.78)	143 (47.67)	516 (49.10)	0.6621
Trimester of first ANC visit	*n*=1,363	*n*=303	*n*=1,060	
1	322 (23.62)	59 (19.47)	263 (24.81)	0.0536*
2	875 (64.20)	198 (65.35)	677 (63.87)	0.6356
3	166 (12.18)	46 (15.18)	120 (11.32)	0.0700
Mode of delivery	*n*=1,369	*n*=306	*n*=1,063	
Spontaneous	1,307 (95.47)	303 (99.02)	1,004 (94.45)	<0.001*
Caesarean	60 (4.38)	3 (0.98)	57 (5.36)	0.001*
Other	2 (0.15)	0 (0.00)	2 (0.19)	0.4454

### Determinants of births in private facilities

Although there was an overall increase in health facility births, from 69.6% at baseline to 77.8% at endline, there was a decrease in private sector deliveries, from 25.1 to 17.3% overall (Table 2). Of the 1,369 women who delivered in a health facility, 22% gave birth in the private sector. Compared to their counterparts who delivered in public health facilities, women delivering in private facilities were significantly more likely to have higher parity, lower socio-economic status, and less education, and were more likely to seek ANC later in pregnancy. They were also more likely to have a normal delivery, associated with the lack of operative capacity in the majority of the private facilities. There was no significant association between the time when women went into labour and the place that they delivered (results not shown).

**Table 2:** Place of delivery

	Baseline		Endline	
	
	*n*=395	%	*n*=1,761	%
Facility delivery	275	69.6	1,369	77.7
Public facility	176	44.6	1,062	60.3
Private facility	99	25.1	306	17.4
Delivered by traditional birth attendant	44	11.1	147	8.3
Delivered at home or elsewhere	110	27.8	245	13.9
Missing	10	2.5	0	0.0

According to the logistical regression analysis (Table 3) women who had two to four previous births compared to those with only one birth were almost twice as likely to deliver in a private health facility (OR 1.86, 95% CI 1.05–3.30). Women with more than four previous births were two times more likely to deliver in a private facility (OR 2.36; 95% confidence interval (CI) 1.34–4.16). In addition, delivery in private health facilities was less likely (although non-significantly so) for mothers of higher wealth quintiles (OR 0.58, CI 0.33–1.02). The odds of women who delivered in private facilities having a caesarean section was 80% lower than in those who delivered in public facilities (OR 0.20, 95% CI 0.48–0.86).

**Table 3:** Determinants of births in private health facilities

Variable	Univariate unadjusted		Multivariate unadjusted	
	
	OR	95% CI	OR	95% CI
Parity				
1	1		1	
2–4	1.72	1.15–2.56	1.72	1.06–2.81
>4	2.22	1.50–3.32	2.01	1.22–3.31
Wealth quintile				
1 (Poorest)	1			
2 (Poor)	0.73	0.46–1.16	0.75	0.46–1.21
3 (Average)	0.76	0.49–1.19	0.75	0.48–1.19
4 (Rich)	0.71	0.44–1.14	0.75	0.46–1.22
5 (Richest)	0.44	0.26–0.73	0.52	0.30–0.90
Education level				
No education	1			
Primary	0.96	0.63–1.48	1.08	0.67–1.76
Secondary or higher	0.56	0.35–0.89	0.93	0.53–1.66
Trimester of first ANC visit				
1	1			
2	1.30	0.94–1.80	1.31	0.89–1.92
3	1.71	1.10–2.66	1.77	1.07–2.95
Mode of delivery				
Normal	1			
Caesarean	0.17	0.05–0.56	0.20	0.48–0.84

### Coverage of essential newborn care practices by place of birth

Amongst all facility births, coverage of essential newborn care practices varied from a low of 58.6% of women practicing dry cord care to 94.8% use of a clean instrument to clamp or tie the umbilical cord. However, the composite indicators of babies receiving all basic essential interventions were much lower, range being 11.5% optimal feeding practices, 46.9% hygienic cord care, and 66.3% receiving thermal protection overall. With the exception of immediate breastfeeding, the coverage of individual essential newborn care practices was higher but not significantly different in public sector facilities compared to private facilities (Table 4). Similarly, the composite essential newborn care indicators of optimal feeding practices, hygienic cord care, and thermal protection were all higher in public facilities, with thermal care practices significantly higher at 68.1% coverage in public sector facilities compared to 59.8% in private facilities (Fig. 2).

**Table 4:** Reported neonatal care practices by place of delivery

	Public health facilities	Private health facilities	
	
Practices	*N* (%)	*N* (%)	*p*
Clean instrument used to cut the cord	*n*=1,063	*n*=306	
	883 (83.07)	252 (82.35)	0.7681
Clean instrument used to tie or clamp the cord	*n*=1,063	*n*=306	
	1,014 (95.39)	285 (93.14)	0.1153
Dry cord care	*n*=1,062	*n*=304	
	631 (59.42)	170 (55.92)	0.2733
Breastfed within first hour	*n*=1,063	*n*=306	
	734 (69.05)	218 (71.24)	0.4633
Baby fed by breast only (no bottle)	*n*=1,063	*n*=306	
	1,026 (96.52)	300 (98.04)	0.1791
Exclusive breastfeeding in the first month	*n*=1,063	*n*=305	
	862 (81.09)	247 (80.98)	0.9655
Baby wrapped after delivery with dry cloth	*n*=1,063	*n*=306	
	1,060 (99.72)	304 (99.35)	0.3428
Baby placed skin-to-skin	*n*=1,063	*n*=306	
	821 (77.23)	218 (71.24)	0.0309*
First bath delayed >6 h	*n*=1,055	*n*=302	
	920 (87.20)	255 (84.44)	0.2146
Hygienic cord care	*n*=1,063	*n*=306	
Clean instrument used to cut cord; clean instrument used to tie cord; nothing placed on cord	502(47.22)	140(45.75)	0.6498
Thermal protection	*n*=1,063	*n*=306	
Baby wrapped after delivery with dry cloth; placed skin-to-skin; bath delayed >6 h	724 (68.11)	183 (59.80)	0.0067*
Optimal feeding practices	*n*=1,063	*n*=306	
Baby breastfed within first hour; no bottle used; exclusive breastfeeding for the first month	128 (12.04)	30 (9.80)	0.2798

Babies born in public health facilities were more likely to receive more individual newborn care practices compared to their private health facility counterparts. Whereas 42.8% of babies born in public facilities received at least eight essential newborn care practices, only 27.5% in private facilities received the same number. Nearly all (98%) babies born in public health facilities received at least three practices, compared to 95% amongst those in private health facilities (Table 5).

**Table 5:** Number and distribution of essential newborn care interventions by place of delivery (%)

	Number of newborn care interventions received										

	9	8	7	6	5	4	3	2	1	0	Mean (SD)
Private facility	8.5	27.4	46.8	68.8	81.0	89.5	95.1	98.2	99.5	100.0	6.16 (1.92)
Public facility	16.4	42.8	67.8	82.9	92.4	96.6	98.4	99.5	99.8	100.0	7.04 (1.59)
*p*											<0.001*

Interventions include: Wrapping the baby using a dry cloth; early skin-to-skin placement; delayed bath at least 6 h after delivery; clean instrument used to cut the umbilical cord; clean device used to tie or clamp the cord; placing nothing on the cord stump; breastfeeding within the first hour after birth; no use of bottle; not giving any food or drink other than breast milk for the first month.

## Discussion

To our knowledge this is the first study to compare the difference in newborn care practices between private and public health facilities in Uganda. We found that there is little difference in newborn care practices in private and public facilities. Private facilities are more likely to be accessed by the poorest families and at-risk women. After health system strengthening, including health worker training, provision of essential supplies, and supervision in both public and private sectors, there was an overall increase in health facility births. During the same period we observed a decline in private sector deliveries.

The increase in facility deliveries in public facilities suggests that the health system strengthening activities had a positive impact on utilisation. Although we cannot make a causal interference as the study is based on two subsequent cross-sectional surveys without any comparison area, the temporal relationship gives some indication that such an association might exist. No other intervention which might confound the association was ongoing in the study area. As the place of delivery is likely to be an indicator which is easily remembered, we do not think that the difference in recall period (4 months at baseline and 12 months at endline) may bias the results; in most surveys recall periods of one year or more are used (39).

Thus, we think that it is likely that the combined supply- and demand-side interventions in UNEST resulted in perceived or real improvements in care, including public sector health workers being more receptive and responsive to clients. Possibly the intervention could have influenced perceived quality of and access to public sector services.

These findings have public health implications for Uganda, and also for other low- and middle-income countries looking to strengthen care at the time of birth.

In this setting, women who delivered at private health facilities had a higher-risk birth profile compared to women who sought care at public facilities. Women who gave birth in private facilities were associated with lower socio-economic status, higher parity, lower education, and were more likely to attend ANC later in pregnancy. Private facility-based delivery care in sub-Saharan Africa is typically associated with the urban rich and more educated segment of the population. However, in our experience, this is only true for the more advanced private health units, not for the majority of private units in rural areas, that are often small and designed to serve the poor.

The use of a private health facility, however, bears the risks of potentially catastrophic costs associated with obstetric care (40, 41), making the pattern of private sector care seeking amongst poorer families potentially harmful. However, a similar pattern as observed in our study in rural Uganda has also been described in Nairobi's informal settlements (31). A plausible explanation could lie in the fact that public health facilities are more distant whereas private care providers are strategically placed to maximise access and to fill a demand gap, especially in more rural areas. In addition, informal payments – which are common in the public sector in Uganda – might have led families to make a rational choice to save on transport costs but pay more for the delivery care (42, 43).

Private health care was not found to be synonymous with better capacity and quality. The private health facilities in the study setting had less capacity in terms of infrastructure, staffing, equipment, and medicines (32). The lack of emergency obstetric services at private health facilities and referral delays pose a real risk to the survival and health of mothers and babies. Through UNEST, private as well as government health facilities were targeted for health system strengthening, with the knowledge that the private ones are often overlooked by development partners and districts in capacity-building efforts such as dissemination of service guidelines; in-service training; provision of equipment, drugs, and supplies; and supervision and mentorship. Although public health facilities performed on par with private health facilities across almost all newborn care practices, coverage overall was not optimal, especially in the case of early and exclusive breastfeeding. These essential newborn care practices are inexpensive and require little if any technology and commodities. The low coverage represents a missed opportunity for all births, regardless of place of delivery.

More information is needed to understand the patterns of care seeking in the public and private sector. There are few disaggregated data available in terms of utilisation as the main source of population-based data, the Demographic and Health Surveys, combines private for-profit and private not-for-profit facilities together, obscuring this potentially important distinction in healthcare provision (9).

This study has some limitations. Newborn care practices were assessed by asking the mother about the care she had received or provided to her newborn. Such responses are subject to recall bias, as some women might not remember interventions implemented during or immediately after birth, particularly for complicated deliveries. However, it can be assumed that such recall bias is similar in women receiving care from a public or private provider. Restricting survey respondents to women who had live births may limit understanding of the potentially different profile of care received by women who experienced stillbirths or early pregnancy loss. However, it was not considered appropriate or feasible to interview such women due to the sensitive nature of their experience. The study did not assess the capacity of private or government health facilities to provide essential newborn care, such as the availability of staff, training received on newborn care, or availability of equipment and supplies. Observation studies would have provided more accurate data, but they consume much time and resources.

## Conclusion

As countries increase attention on improving coverage and quality of maternal and newborn care, and in the context of the post-Millennium Development Goal agenda focusing on universal health care, it is important to also consider the role of private sector providers, especially those in rural and urban poor areas which serve under-reached, vulnerable populations. Financial, geographic, and sociocultural barriers to accessing public sector care should be further explored. As for the public facilities, the private sector also requires accountability mechanisms and capacity-building activities, including training and supervision support and guidance on evidence-based best practices for newborn care.
